# Improved statistical model checking methods for pathway analysis

**DOI:** 10.1186/1471-2105-13-S17-S15

**Published:** 2012-12-07

**Authors:** Chuan Hock Koh, Sucheendra K Palaniappan, PS Thiagarajan, Limsoon Wong

**Affiliations:** 1 NUS Graduate School for Integrative Sciences and Engineering, Singapore 117597; 2 School of Computing, National University of Singapore, Singapore 117417

## Abstract

Statistical model checking techniques have been shown to be effective for approximate model checking on large stochastic systems, where explicit representation of the state space is impractical. Importantly, these techniques ensure the validity of results with statistical guarantees on errors. There is an increasing interest in these classes of algorithms in computational systems biology since analysis using traditional model checking techniques does not scale well. In this context, we present two improvements to existing statistical model checking algorithms. Firstly, we construct an algorithm which removes the need of the user to define the *indifference region*, a critical parameter in previous sequential hypothesis testing algorithms. Secondly, we extend the algorithm to account for the case when there may be a limit on the computational resources that can be spent on verifying a property; i.e, if the original algorithm is not able to make a decision even after consuming the available amount of resources, we resort to a p-value based approach to make a decision. We demonstrate the improvements achieved by our algorithms in comparison to current algorithms first with a straightforward yet representative example, followed by a real biological model on cell fate of gustatory neurons with microRNAs.

## Introduction

Model checking is an automated method to formally verify a system's behavior. It is a technique widely used to validate logic circuits, communication protocols and software drivers [1]. Usually, the system to be analyzed is encoded in a specification language suitable for automated exploration, and the properties to be verified are specified as formulas in temporal logics. Given a model of the system and a temporal logic formula, the model checker systematically explores the state space of the model to check if the specified property is satisfied. If the property holds, the model checker returns the value *true*; otherwise, the model checker returns a *false *value, with a counter example of a specific trace of the system where the property failed.

Recently there have been efforts to apply model checking in computational systems biology [2-7]. In this context, probabilistic models -- such as Discrete Time Markov Chain (DTMC) or Continuous Time Markov Chain (CTMC) -- are often used and, properties are expressed with specialized probabilistic temporal logics that quantify the properties with probability. We refer to this as *probabilistic model checking*.

Usually probabilistic model checking is solved using numerical solution techniques, and typically involves iteratively computing the exact probability of paths satisfying appropriate sub-formulas. There are several efficient optimizations to represent the state space of these models compactly, and to traverse the state space efficiently. However, they are usually very memory intensive and do not scale well to large stochastic models. Hence, approximate methods for solving such problems are often used. One such class of methods, known as *statistical model checking*, relies on using, as the name suggests, statistical techniques to perform model checking. It is based on simulating a number of sample runs of the system and, subsequently, deciding whether the samples provide enough evidence to suggest the validity or invalidity of the property specified as a probabilistic temporal logic formula [8].

Statistical model checking is based on the crucial observation that it may not be necessary to obtain an absolute accurate estimate of a probability in order to verify probabilistic properties. For example, to verify if the probability of a random variable exhibiting a certain behavior is greater than *θ*, it is not necessary to compute the exact probability of the property (*p*) to hold; instead, it is enough if we infer, by sufficiently sampling the underlying model, that the probability is safely above or below *θ*. Approaches based on statistical model checking are proven to be scalable, since they are not dependent on constructing and traversing the full state space of the model. Additionally, they have a low time complexity, require low memory, and are tunable to the desired accuracy. These factors make them ideal for performing analysis on large complex stochastic systems.

Since computational pathway models are typically large complex stochastic models, our focus in this paper is on the statistical model checking problem. A standard version of statistical model checking, which is the one we focus on in this paper, is called sequential hypothesis testing [9,10]. The success of this approach depends largely on a user-defined parameter called the indifference region. The choice of the indifference region dictates the number of samples necessary to verify the property and the outcome of the verification task. Consequently, it will be helpful to have a method of specifying the indifference region that does not solely depend on the user-input.

Furthermore, when the true probability of the property is very close to the probability specified in the formulas, a large number of simulations is needed to validate or invalidate the property. Maintaining an optimal balance between computational effort and precision is important. It may well be the case with existing algorithms that, to satisfy the specified error bounds, a large number of samples are drawn. In such cases, it will be useful to return a reasonable answer once a pre-specified amount of computational resources have been consumed while the statistical test required is unable to make a decision yet.

To address these issues, we propose optimized sequential hypothesis testing algorithms which 1) do not need the user to provide the indifference region parameter; 2) adjusts to the difficulty of the problem, i.e. the distance between *p *(the true probability) and *θ *(probability dictated by the property) dynamically; 3) always provides a definite *true *or *false *result, i.e, does not return the undesirable *undecided *result (or "I do not know" response).

### Related work

Existing works on statistical model checking can be classified based on whether the probabilistic system is a black-box or a white-box system. A white-box system allows generation of as many trajectories of the system as desired. In a black-box system, only a fixed number of trajectories is available and, using which a decision has to be made. We establish the basic concepts and terminologies to be used in the rest of the paper in this section. Formally, probabilistic model checking refers to the problem of verifying if *M *= *Pr_Δθ_*{*ψ*}, Δ ∈ {≤, ≥, >, <}; i.e, given a probabilistic model *M*, and a property *ψ *encoded in a probabilistic temporal logic formalism, check whether *ψ *holds in *M *with probability dictated by Δ w.r.t to *θ*.

### Black-box systems

Statistical model checking on black-box systems is based on calculating a p-value that quantifies the statistical evidence of satisfaction or rejection of a hypothesis using the set of samples given [11,12]. Sen *et al *gives an algorithm for black box systems which quantifies the evidence of satisfaction of the formula by a p-value [11]. The problem is formulated as solving two separate hypothesis tests (*H*0: *p *<*θ *against *H*1: *p *≥ *θ*). If ∑ *x_i_/n *≥ *θ *(where *x_i _*is 1 if the *i*th sample satisfies *ψ *and 0 if it does not), *H*0 is rejected, the formula is declared to hold, and the p-value is calculated. If the test does not reject *H*0 then a second experiment is conducted, with *H*0: *p *≥ *θ *against *H*1: *p < θ*. If ∑ *x_i_/n < θ, H*0 is rejected, the formula is declared *false*, and the corresponding p-value is calculated. The smaller the p-value, the greater is the confidence in the decision.

Younes also discusses an algorithm for black box systems using a modified version of single-sampling plan with p-value [13]. Younes proposes the single-sampling-based hypothesis testing algorithm where the number of samples *n *is decided upfront. The model checking problem is formulated as a hypothesis test with the null hypothesis *H*0: *p *≥ *θ *against the alternate hypothesis *H*1: *p < θ*, a constant *c *is also specified that decides the number of samples that should evaluate to *true *to accept the null hypotheses. Let *X_i _*be a Bernoulli random variable with parameter *p *such that *Pr*[*X_i _*= 1] = *p *and *Pr*[*X_i _*= 0] = 1 - *p*. An observation/sample of *X_i_*, represented as *x_i_*, states whether the specified temporal logic formula is *true *or *false *for a particular observation. For example, in this case, *x_i _*is 1 if the *i*th sample satisfies *ψ *and 0 if it does not. The *strength *of the hypothesis test is decided by parameters *α *and *β*, which represent the probability of false negatives (Type-1 error) and false positives (Type-2 error) respectively. If $\sum_{i=1}^{n}x_{i}>c$ then the hypothesis *H*0 is accepted; else *H*1 is accepted. The main challenge is to find the pair 〈*n, c*〉 which obey the error bounds 〈*α, β*〉. Younes describes an algorithm based on binary search to find the pair 〈*n, c*〉 that obeys the bounds [13].

### White-box systems

Model checking on white-box systems can be classified into those which are based on either statistical estimation or hypothesis testing. Statistical estimation based methods rely on getting an estimate of the true probability, *p*, and comparing it with *θ *(dictated by the temporal logic formula) to make a decision [14]. Algorithms based on hypothesis testing formulate the model checking problem into a standard hypothesis test between a null and alternate hypothesis. Using techniques developed for solving hypothesis testing problems, a decision is made about the satisfiability of the property. Methods based on hypothesis testing can be further subdivided into two different approaches -- those that rely on *Frequentist *statistical procedures [9,10]; and those that use *Bayesian *statistical procedures [15,16].

Bayesian methods have the advantages of smaller expected sample sizes and ability to incorporate prior information. However, Bayesian methods are generally more computationally expensive than their frequentist counterpart due to the requirement to produce a posterior distribution [17]. In Bayesian methods, the degree of confidence is indicated via a parameter called, *Bayes factor threshold*, whereas frequentist methods use error bounds (Type-1 (*α*) and Type-2 (*β*) error). To say one is better than the other would be going into the old debate between Frequentist and Bayesian statistics. However, we prefer the frequentist approach since it allow us to explicitly state the error bounds, which is more intuitive to us.

### Frequentist statistical model checking

**Figure 1:**
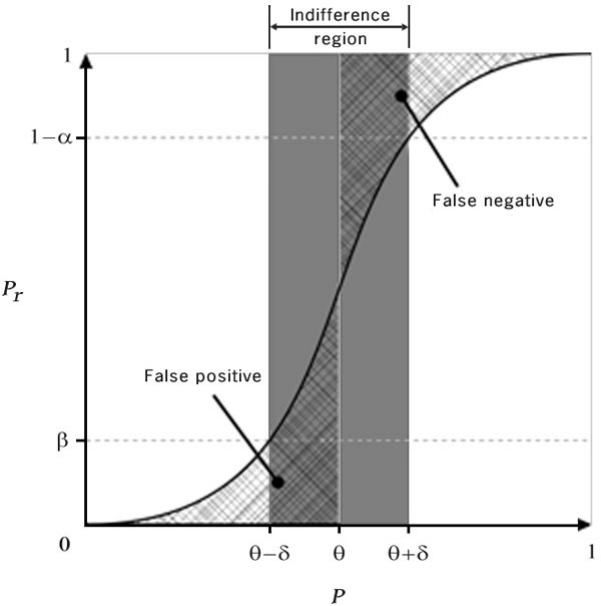
**Probability of accepting *H0 *: *p *≥ *θ *+ *δ *vs the actual probability of the formula holding (adapted from **[13]).

Younes and Simmons formulate the probabilistic model-checking problem as a sequential hypothesis-testing problem. Here, we call their algorithm Younes A [9], which is as follows: To verify a formula of the form *Pr*_≥*θ *_{*ψ*}, a hypothesis test is setup between a null hypothesis *H*0: *p *≥ *θ *+ *δ *against the alternative hypothesis *H*1: *p < θ - δ*. The factor *δ *represents the indifference region around the threshold *θ*. This is represented in Figure 1. Algorithms based on sequential hypothesis testing need input parameters *α, β, δ *which specify the Type-1, Type-2 error bounds and the indifference region respectively. These parameters help in controlling the number of samples and guaranteeing the desired error rates. For a fixed value of *α *and *β, δ *decides the number of samples needed to verify a property. It is inversely proportional to the number of samples required; i.e., the smaller *δ *is, the more samples are needed. Also, the smaller *δ *is, the lesser is the probability of *p *being in the region [*θ *- *δ, θ *+ *δ*]. *δ *is a user-defined parameter whose choice is problem specific and usually involves iterative tuning. Hence, deciding the optimal value of *δ *affects the practical applicability of these algorithms. A sequential sampling algorithm based on Wald's sequential probability test is used to solve the hypothesis testing problem. After taking the *n^th ^*sample from the model, the factor *f_n _*is calculated as,

(1) $$f_{n}=\overset{n}{\underset{i=1}{\prod }}\frac{Pr\left[X_{i}=x_{i}|p=\theta -\delta \right]}{Pr\left[X_{i}=x_{i}|p=\theta +\delta \right]}=\frac{{\left[\theta -\delta \right]}^{\left(\sum_{i=1}^{n}x_{i}\right)}{\left[1-\left[\theta -\delta \right]\right]}^{\left(n-\sum_{i=1}^{n}x_{i}\right)}}{{\left[\theta +\delta \right]}^{\left(\sum_{i=1}^{n}x_{i}\right)}{\left[1-\left[\theta +\delta \right]\right]}^{\left(n-\sum_{i=1}^{n}x_{i}\right)}}$$

Hypothesis *H*0 is accepted if *f_n _*≤ *B*; Hypothesis *H*1 is accepted if *f_n _*≥ *A*; and if *B < f_n _< A*, another sample is drawn. The constants *A *and *B *are chosen so that they result in a test of strength 〈*α, β*〉. In practice, to satisfy the strength dictated by 〈*α, β*〉 choose $A=\frac{1-\beta }{\alpha }$ and $B=\frac{\beta }{1-\alpha }$. The algorithm satisfies the error bounds 〈*α, β*〉 only when the true probability does not lie in the indifference region, which is an issue.

To address this issue, Younes discusses a modified algorithm (Algorithm 1), which we call Younes B here, that bounds the error for cases when the true probability lies in the indifference region by introducing a factor (*γ*), which allows and controls the probability of *undecided *results (when the true probability is within the indifference region) [10]. Younes B uses two acceptance sampling tests:

$$\begin{array}{c} H0:p\geq \theta \;against\;H1:p<\theta -\delta \;with\;strength\;\;\left\langle \alpha ,\gamma \right\rangle \\ H0^{\prime }:p\geq \theta \;+\delta \;against\;H1^{\prime }:p<\theta \;with\;strength\;\;\left\langle \gamma ,\beta \right\rangle \end{array}$$

*Pr*_≥*θ*_{*ψ*} is reported as *true *when both *H*0 and *H*0' are accepted. It is reported as *false *when both *H*1 and *H*1' are accepted. Otherwise, the results is reported as *undecided*. It is not meaningful to distinguish between *Pr*_>*θ*_{*ψ*} and *Pr*_≥*θ*_{*ψ*}; and *Pr*_>*θ*_{*ψ*} can essentially be written as *Pr*_≥*θ*_{*ψ*}. Therefore, it is sufficient to present on this case, *Pr*_≥*θ*_{*ψ*}.

**Algorithm 1 **ORIGINAL YOUNES B (*θ, δ, α, β, γ*)

$$\begin{array}{c} n\leftarrow 0,d\leftarrow 0;\\ \begin{array}{cc} A_{1}\leftarrow \text{log}\frac{1-\gamma }{\alpha }; & \left\{Accept\;H1\;\;if\;f_{n}>A_{1}\right\} \end{array}\\ \begin{array}{cc} B_{1}\leftarrow \text{log}\frac{\gamma }{1-\alpha } & \left\{Accept\;H0\;\;if\;f_{n}<B_{1}\right\} \end{array}\\ \begin{array}{cc} A_{2}\leftarrow \text{log}\frac{1-\beta }{\gamma }; & \left\{Accept\;H1\prime \;\;if\;f_{n}^{\prime }>A_{2}\right\} \end{array}\\ \begin{array}{cc} B_{2}\leftarrow \text{log}\frac{\beta }{1-\gamma }; & \left\{Accept\;H0\prime \;\;if\;f_{n}^{\prime }<B_{2}\right\} \end{array} \end{array}$$

repeat

   *n *← *n + *1; {Generates a new sample}

   **if **(*x_n _*= = 1) **then**

      *d *← *d *+1;

   **end if**

$$\begin{array}{c} f_{n}\leftarrow d\text{log}\frac{\theta -\delta }{\theta }+\left(n-d\right)\text{log}\frac{1-\left(\theta -\delta \right)}{1-\theta };\\ f_{n}^{\prime }\leftarrow d\text{log}\frac{\theta }{\theta +\delta }+\left(n-d\right)\text{log}\frac{1-\theta }{1-\left(\theta +\delta \right)}; \end{array}$$

   **until! **$\left(\left(B_{1}<f_{n}<A_{1}\right)||\left(B_{2}<f_{n}^{\prime }<A_{2}\right)\right)$

   **if **$\left(\left(f_{n}<B_{1}\right)\&\&\left(f_{n}^{\prime }<B_{2}\right)\right)$**then**

      Return *TRUE*; {*p *≥ *θ*}

   **else if **$\left(\left(f_{n}>A_{1}\right)\&\&\left(f_{n}^{\prime }>A_{2}\right)\right)$**then**

      Return *FALSE*; {*p *≱ *θ*}

   **else**

      Return *UNDECIDED*;

   **end if**

   *x_n _***is the outcome of the ***n***th sample**, 1 **if ***true ***else **0

*α ***is the Type I error**

*β ***is the Type II error**

## Optimized Statistical Model checking algorithm (OSM)

As discussed earlier, we aim to remove the manual selection of the indifference region parameter. The rationale behind this is because, while the parameter is critical to the success of previous sequential hypothesis testing algorithms, it is very difficult for the user to select a suitable value. We combine ideas from the realms of verifying white box and black box to produce an algorithm that is practically superior. The essence of our proposed algorithms is similar to Younes B's two-acceptance-sampling-tests approach but, we make several critical changes which enhance them significantly. We describe our algorithm in the following subsections.

### Adjusting *δ *automatically

Instead of having to specify a difficult-to-determine indifference region (explained in detail later), we first assume it to be 1:0, which is the largest possible value. We start with a large *δ *because, the larger *δ *is, the fewer samples we need. We then proceed with using two simultaneous acceptance-sampling tests just like [10]. However, the crucial difference is that, whenever an *undecided *result is returned by the algorithm, we reduce *δ *by half and check whether 1) a definite result can be given, 2) another sample is needed, or 3) a further reduction is required. We continue this process until a definite result is produced. The details are given in Algorithm 2.

**Algorithm 2 **OSM A (*θ, α, β*)

*δ *← 1, *γ *← *min *(*α, β*), *n *← 0, *d *← 0;

**while **(*true*) **do**

   (*n, d, y*) ← *Incremental Younes B*(*θ, δ, α, β, γ, n, d*);

   **if **((*y *= = *TRUE*) || (*y *= = *FALSE*)) **then**

      Return *y*;

   **else**

      *δ *← *δ*/2; {Undecided with current *δ*, halve it}

   **end if**

end while

**Function***Incremental Younes B*(*θ, δ, α, β, γ, n, d*);

$$\begin{array}{c} \begin{array}{cc} A_{1}\leftarrow \text{log}\frac{1-\gamma }{\alpha }; & \left\{\text{Accept}\;\text{H}1\;\;if\;f_{n}>A_{1}\right\} \end{array}\\ \begin{array}{cc} B_{1}\leftarrow \text{log}\frac{\gamma }{1-\alpha } & \left\{\text{Accept}\;\text{H}0\;\;if\;f_{n}<B_{1}\right\} \end{array}\\ \begin{array}{cc} A_{2}\leftarrow \text{log}\frac{1-\beta }{\gamma }; & \left\{\text{Accept}\;\text{H}1\prime \;\;if\;f_{n}^{\prime }>A_{2}\right\} \end{array}\\ \begin{array}{cc} B_{2}\leftarrow \text{log}\frac{\beta }{1-\gamma }; & \left\{\text{Accept}\;\text{H}0\prime \;\;if\;f_{n}^{\prime }<B_{2}\right\} \end{array} \end{array}$$

repeat

   *n *← *n + *1; {Generates a new sample}

   **if **(*x_n _*= = 1) **then**

      *d *← *d *+1;

   **end if**

$$\begin{array}{c} f_{n}\leftarrow d\text{log}\frac{\theta -\delta }{\theta }+\left(n-d\right)\text{log}\frac{1-\left(\theta -\delta \right)}{1-\theta };\\ f_{n}^{\prime }\leftarrow d\text{log}\frac{\theta }{\theta +\delta }+\left(n-d\right)\text{log}\frac{1-\theta }{1-\left(\theta +\delta \right)}; \end{array}$$

   **until ! **$\left(\left(B_{1}<f_{n}<A_{1}\right)||\left(B_{2}<f_{n}^{\prime }<A_{2}\right)\right)$

   **if **$\left(\left(f_{n}<B_{1}\right)\&\&\left(f_{n}^{\prime }<B_{2}\right)\right)$**then**

      Return (*n, d, TRUE*); {*p *≥ *θ*}

   **else if **$\left(\left(f_{n}>A_{1}\right)\&\&\left(f_{n}^{\prime }>A_{2}\right)\right)$**then**

      Return (*n, d, FALSE*); {*p *≱ *θ*}

   **else**

      Return (*n, d, UNDECIDED*);

   **end if**

   *x_n _***is the outcome of the ***n***th sample**, 1 **if ***true ***else **0

   *α ***is the Type I error**

   *β ***is the Type I error**

   *n ***is the number of samples**

   *d ***is the number of samples satisfying ***ψ*

Our algorithm (OSM A) has three advantages over previous works. First, a predetermined user-defined indifference region *δ *is not required. Secondly, the number of samples required adjusts automatically to the difficulty of the problem, i.e., depending on how close *p *is to *θ*, by starting with the largest possible indifference region. Finally, our algorithm always gives a definite result if sufficient samples are given and, that result is guaranteed to be error bounded.

However, if *p *is very close to *θ*, the indifference region needs to be reduced to a very small value such that *δ *< |*p *- *θ*|. If *δ *is very small, the sample size required to determine a result will be very large or, in the worst case where *p *= *θ*, this algorithm will not terminate. Therefore, while such an algorithm is superior in theory, it may be limited in some situations in practice. Hence, in sub-section, we further improve this algorithm by setting a limit on the sample size. This will ensure that the program completes in a user-acceptable runtime to handle such unlikely but possible situations.

The ability of OSM A to control errors is obviously dependent on Younes B algorithm's ability to control them. Therefore, interested readers are referred to [10] where they provide proofs for the strength of two acceptance sampling tests. In this paper, we empirically demonstrate in section that OSM A consistently has the ability to control errors in various settings.

**Figure 2:**
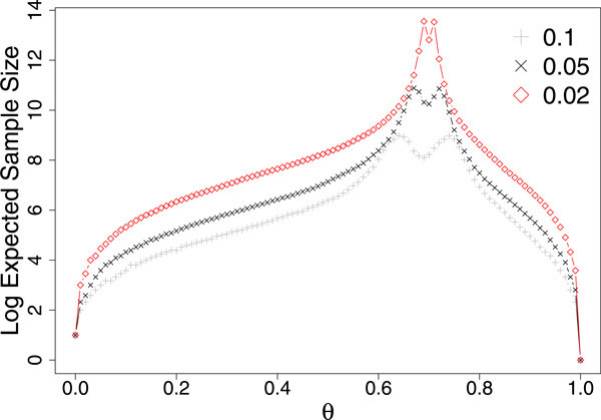
***Log*_2 _expected value of n at which the Younes B algorithm terminates and returns a TRUE/FALSE/UNDECIDED answer for different values of *δ *(0.02, 0.05 and 0.1) at varying *θ *with *α *= 0.01, *β *= 0.01, *γ *= 0.01 and *p *= 0.7**. This figure demonstrates that with decreasing *δ*, the expected *n *increases.

Based on Algorithm 2, as OSM A repeatedly calls Incremental Younes B, it would require much more samples to be generated than Younes B. However, that is not true. This is because OSM A reuses samples from previous iterations (with a different *δ*) instead of starting from scratch with each call. Therefore, the number of samples needed to be generated by OSM A is actually the same as Younes B given the same *θ, α, β, γ *and *δ*. It is possible to reuse samples from different iterations because, given the same *θ, α, β*, and *γ *if the Younes B algorithm running at a larger value of *δ *terminated at a value of *n *but returned UNDECIDED, then the Younes B algorithm running at a smaller value of *δ *would not terminate and return TRUE/FALSE at that same value of *n *(though it would terminate at a higher value of *n *and return a TRUE/FALSE/UNDECIDED answer) (Figure 2).

### Limiting the number of samples

By limiting the sample size, we can bound the runtime of the program but we may not be able to bound the error rates. Therefore, we compute a p-value to serve as a measure of the confidence of the result. The modified algorithm is as follows. As before, we first assume *δ *to be the largest possible, i.e., 1:0. Then we proceed using two simultaneous acceptance-sampling tests and, whenever an undecided result is given, we reduce *δ *by half and check whether 1) a definite result can be given, 2) another sample is needed, or 3) a further reduction is required. We continue this process until a definite result is given or when the sample size limit is reached. If a definite result is reached before the sample size limit, then the error rate is guaranteed to be bounded. Otherwise, if the sample size limit is reached, we compute the p-value for both hypotheses *H*0: *p *≥ *θ *and *H*1: *p < θ*, and accept the hypothesis with the lower p-value. The p-values are computed using the method presented in [12] -- viz., the p-value for *H*0 is 1 - *F*(*d*; *n, θ*) and the p-value for *H*1 is *F*(*d*; *n, θ*), where *d *is the number of successes (or true), *n *is the total number of samples, and *F*(*d*; *n, θ*) is the Binomial cumulative distribution function,

(2) $$F\left(d;n,\theta \right)=\overset{d}{\underset{i=0}{\sum }}\left(\begin{array}{c} n\\ i \end{array}\right)\theta^{i}{\left(1-\theta \right)}^{n-1}$$

With this, we have developed an algorithm that 1) does not require the user to predetermine a suitable indifference region, 2) is guaranteed to bound specified Type-1 and Type-2 errors if sufficient samples can be generated, and 3) terminates and returns a confidence measure even in the rare event when *p *is extremely close to or equal to *θ*. We call the above algorithm OSM B.

In the next section, we demonstrate the superiority of our proposed algorithms against current state-of-art, first with a straightforward yet representative example followed by applying to a real biological model.

## Results

For a fair comparison across different algorithms, we need to define the performance measures of interest. In model checking, simulation runs are typically the most computationally expensive and obtaining accurate conclusions about the model is of paramount importance. Therefore, the most desirable situation would be to obtain accurate conclusions of the model's behavior with the minimum number of simulation runs. As such, we use error rates and simulation runs (or samples) required of each algorithm as the basis for judging superiority in our comparison.

### Simple model

**Figure 3:**
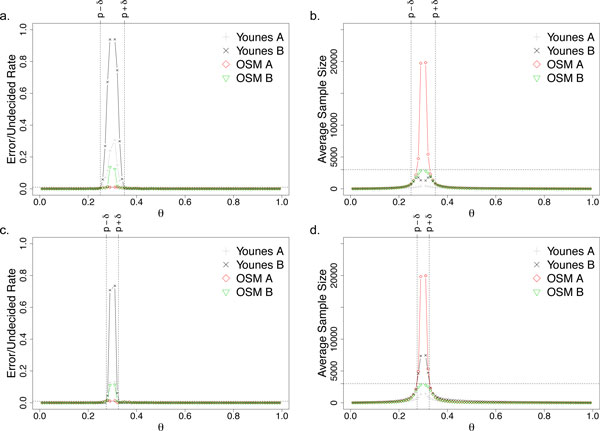
**Plots a & b are with an indifference region of 0.05 whereas c & d are with an indifference region of 0.025 for the small synthetic model**.

Here, we use a simple uniform random generator that produces real numbers in the range of [0, 1] as our probabilistic simulation model. Suppose the property that we are testing is whether *p *≥ *θ*, and we fixed *p *(the true probability) to 0.3. To generate a sample, we use the uniform random generator to generate a random number and, the sample is treated as a true sample if and only if the generated value is lesser than *p*. We vary *θ *from [0.01, 0.99] (except *p *which is 0.3) with an interval of 0.01 and set *δ *to be 0.05 and 0.025 for Figure 3a, b and 3c, d respectively. For each setting, the experiments are repeated 1000 times with *α *(Type-1 error rate) and *β *(Type-2 error rate) of 0.01. We also limit the sample size for OSM B to be 3000.

Figure 3 shows how critical and difficult the selection of *δ *is for Younes A and Younes B. Too large, the error and undecided rates within the wide indifference region are unbounded and high (Figure 3a). On the other hand, if *δ *is too small, then the number of samples required grows rapidly in the indifference region (Figure 3d).

Indeed, if a suitable *δ *can be chosen for Younes A and Younes B, the error rate is bounded and minimum samples are used. However, it is a difficult task to choose an ideal *δ *that balances the samples required and the error rates unless one has a good estimate of *p *(the true probability), which is unrealistic.

Furthermore, it should be noted that the Younes A algorithm does not provide information on whether the error rate is bounded or not, i.e., whether *p *is within or outside the indifference region. This implies that the user may come to a false conclusion that the result is bounded with a certain error rate when it is actually not (Figure 3a and 3c).

While Younes B algorithm does indeed always bound the error rate when a definite result is given, it comes at the expense of a large number of undecided results when *p *is inside the indifference region. This means the algorithm uses up computational resources and, in the end, returns an undecided result, which is undesirable.

Our proposed algorithm (OSM A) overcomes all these problems. First, the tough decision of choosing the indifference region is not required as the algorithm does do so dynamically and error rates are always bounded (Figure 3a and 3c). However, OSM A has a limitation in that it requires rapidly increasing number of samples as *θ *closes in on *p *(Figure 3b and 3d).

OSM B removes this limitation by limiting the number of samples and ensures termination (Figure 3b and 3d). We should note that whenever OSM B returns a definite answer, the error is guaranteed to be bounded and, when the sample limit is reached, a confidence measure (p-value) is given. Therefore, it is clear to the user when a result is guaranteed to be error bounded and when it is not.

### Cell fate model of gustatory neurons with microRNAs

**Figure 4:**
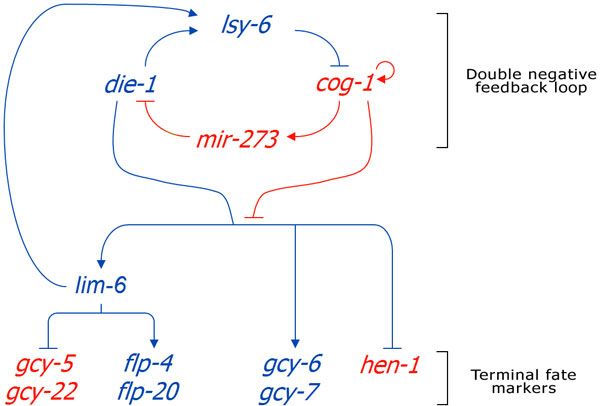
**Summary of the ASE pathway model**. Four regulatory factors lsy-6, cog-1, die-1 and mir-273 form a double-negative feedback loop which determines whether the cells will be ASEL or ASER. In ASEL cells, flp-20, flp-4, gcy-6 and gcy-7 (coded in blue) are expressed, whereas in ASER cells, gcy-5, gcy-22 and hen-1 (coded in red) are expressed [18].

Next, we perform model checking on the cell fate determination model of gustatory neurons of *Caenorhabdities elegans *[18]. This model studies the regulatory aspects mediated by miRNA's on the ASE cell fate in *C.elegans *and focuses on a double negative feedback loop which determines the cell fate (Figure 4). A precursor cell state have equivalent potential to transit into the stable ASEL or ASER terminal state. While ASEL and ASER are physically asymmetric, they are morphologically bilaterally symmetric. It is believed that the cell fate (ASEL or ASER) is controlled by miRNA (*lsy-6 *and *mir-273*) in the double negative feedback loop. The computational model contains 22 entities (RNA or protein) and 27 processes (biological reactions). We first use a property from [7], where it validates that the concentration of LSY-2 in the nucleus will never increase if it has risen and fallen once previously, to illustrate the technical superiority of our proposed algorithms even in real biological examples. We will then discuss its practical implication in the next section.

**Figure 5:**
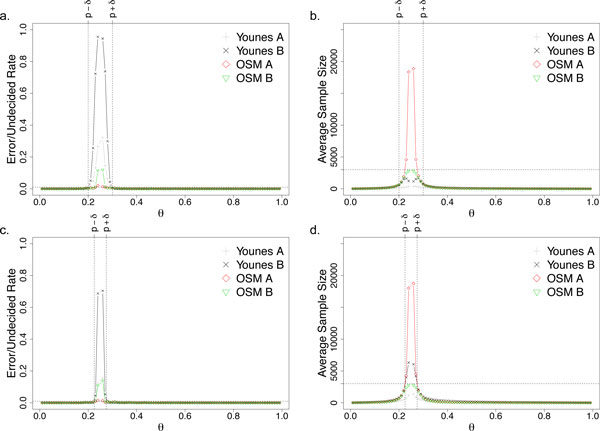
**Plots a & b are with indifference region of 0.05 whereas c & d are with indifference region of 0.025 for the cell fate model**. As expected, this figure looks identical to Figure 3 as these algorithms do not make assumptions on the underlying stochastic model.

As before, we vary *θ *from [0.01, 0.99] (except *p *which is estimated to be 0.25) with an interval of 0.01 and set *δ *to be 0.05 and 0.025 for Figure 5a, b and 5c, d respectively. For each setting, the experiments are repeated 1000 times with fixed *α *(Type-1 error rate) and *β *(Type-2 error rate) of 0.01. We again limit the sample size for OSM B to be 3000. Using a separate experiment of 10,000,000 simulation runs, we had estimated that the true probability, *p*, of this property to be 0.25 (in the 10 million run results, approximately 25% of them satisfied the property).

Figure 5 again demonstrates the superiority of our proposed algorithms over current state-of-the-art. To give an even clearer picture of the advantages of our algorithm, we shall look at the cross-section of a few crucial data points (Table 1).

**Table 1:** Cross-section of Figure 5

		Average Sample Size				Total Errors/Undecided			
***Θ***	***δ***	**Younes A**	**Younes B**	**OSM A**	**OSM B**	**Younes A**	**Younes B**	**OSM A**	**OSM B (P-Value)**

0.5	0.05	45.9	102.5	34.1	34.1	0	0	0	0
					
	0.025	92.0	194.4			0	0		

0.28	0.05	288.8	1560.7	2063.0	1807.6	54	254	5	5
					
	0.025	614.5	2091.4			2	0		

0.26	0.05	393.8	1176.2	18832.7	2784.7	324	937	7	7 (107)
					
	0.025	1316.6	6179.6			129	738		

Cross-section of Figure 5 where *θ *= (0.5, 0.28 and 0.26). At *θ *= 0.26, total errors made by OSM B is 114 out of which 107 is due to p-value (sample limit reached).

Firstly, when *θ *is distant from *p*, the problem is easy. Ideally, algorithms should use minimum amount of samples while maintaining the error bound. In Table 1, at *θ *= 0.5, although all algorithms kept well within the error bounds but Younes A and B both requires much more samples than OSM A and B on average.

As *θ *approaches *p*, understandably more samples would be required to make an accurate conclusion. In these situations, the priority would typically still be to ensure error rates are under control while not using an exorbitant number of samples. Based on Table 1, at *θ *= 0.28, error rate of Younes A and B are dependent on the choice of *δ*. If user is able to choose *δ *to be 0.025, errors are low (Younes A made 2 errors while Younes B made no error) but if user made a wrong choice, *δ *= 0:05, it would be disastrous (Younes A made 54 errors while Younes B made 254 errors/undecided). Since *δ *is not a parameter for OSM A and B, their performance are consistent, with error rates within 1% (or 10 errors in 1000 runs) and average sample size around 2000.

**Figure 6:**
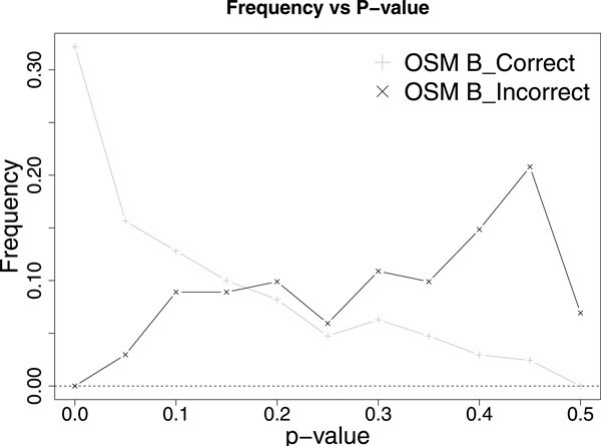
**P-value distribution of OSM B (from Figure 5) when *θ *= 0.26**. Average p-value for OSM B_Correct is 0.147 whereas average p-value for OSM B_Incorrect is 0.357.

In the event where *θ *is extremely close to (or equal) *p*, it is hard (or impossible) to accurately decide unless we have huge (or infinite) samples. Therefore, one could only choose between high accuracy or minimum samples. Our proposed algorithms are useful each in one situation. If high accuracy is desired by the user, OSM A is suitable. As shown in Table 1, *θ *= 0.26, OSM A is constantly keeping errors close to 1% or 10 errors. If computation limitation is of concern to the user, OSM B could be used to maintain sample size limit. Younes A seems to perform better than OSM B, since it uses less samples and have relatively similar errors. However, it is important to note that this is actually not true because, when OSM B could not guarantee the error rate, it returns a p-value (107 errors are made by p-value), instead of the typical *true *or *false *conclusion, which would alert the user to be cautious. In contrasts, Younes A does not have such differentiation and might mislead user to trust its decision. Furthermore, the value of each resulting p-value can be used as another red flag, as OSM B tends to be correct when the p-value is small and incorrect when the p-value is large (Figure 6). As for Younes B, it is even worse, it would run thousands of simulations and give undecided conclusion, which is not very useful, up to 93.7% of the time.

### Practical implications

In the previous few sections, we have shown the superiority of our algorithms from a technical standpoint. In this section, we discuss the practical implications of our algorithms. In particular, we use model checking to verify two behaviors of the ASE pathway model.

#### Equivalent potential to transit into ASER or ASEL

One important application of model checking is using it to ensure that the created simulation model exhibits behaviors that are widely accepted. In literature, it is stated that a precursor ASE cell state should have equivalent potential to transit into stable ASER or ASEL. Therefore, we need to first ensure that the ASE pathway model created exhibits this behavior before we can deem the model to be correctly built and utilize it to perform any downstream analysis.

Suppose we accept equivalent potential to be between 45% and 55%, which means the simulation model should transit into ASER or ASEL with a probability of 0.45 to 0.55. Translating this to model checking language would mean that ASER terminal cell fate markers (such as gcy5) should be more abundant than ASEL terminal cell fate markers (such as gcy6) after some simulation time with a probability of 0.45 to 0.55. More formally, the *PLTLs *format would be *P *_≥ 0.45_*G*([*gcy*5] > [*gcy*6]) {[*time*] > 300} AND *P *_≤ 0.55_*G*([*gcy*5] > [*gcy*6]) {[*time*] > 300}. Readers unfamiliar with temporal logics and model checking in systems biology can find the relevant background materials in [19-21].

By using a separate, computationally expensive experiment of 10,000,000 simulation runs, we found that the ASE model transits into ASER and ASEL with approximately 46% and 54% probability respectively. Therefore, the correct conclusion to be given by the algorithms should be to accept that the model as correct.

Assuming that a user wants the error rate to be around 1% (*α *= 0.01 *β *= 0.01), and has chosen *δ *to be 0.025. Table 2 shows that there is a 13.1% probability that Younes A incorrectly rejects the model while there is a 70.3% probability that Younes B replies with an *undecided *response.

**Table 2:** Verifying ASE model

		Average Sample Size					Total Errors/Undecided		
**Δ**	***θ***	**Younes A**	**Younes B**	**OSM A**	**OSM B**	**Younes A**	**Younes B**	**OSM A**	**OSM B (P-Value)**

≥	0.45	1593.1	8469.2	23769.5	2810.0	131	703	12	12 (114)

≤	0.55	262.1	595.8	311.2	311.2	0	0	0	0

To verify whether the ASE model has equivalent potential to transit into ASER or ASEL. With *α *= 0.01, *β *= 0.01, *γ *= 0.01, *δ *= 0.025 and repeating the experiment 1000 times.

On the other hand, as shown in Table 2, OSM A only gives a wrong conclusion with 1.2% probability. However, OSM A requires, on average, ≥ 23,000 simulation runs to make a decision, which could be much more than the available computational resources to the user. In such cases, the user can still depend on OSM B where it needs only about 2,800 simulation runs on average, with only 1.2% probability of giving a wrong conclusion. The rest of the 11.4% wrong decisions given by OSM B is when computational resources are maxed out and OSM B returns a p-value instead of the *true *or *false *response. This should alert the user to be more cautious of the conclusion.

#### lsy-2 in the nucleus will never increase if it has risen and fallen once previously

Suppose that after validating the model, we are now interested in investigating whether the ASE model exhibits the following behavior: There is more than 28% probability that the concentration of lsy-2 in the nucleus will never increase if it has risen and fallen once previously. Translating this to *PLTLs *would be *P *_≥ 0.28_((*d*([*lsy*2*N *]) > 0) *U*(*G*(*d*([*lsy*2*N*]) ≤ 0))).

Once again, by using a computationally expensive, separate experiment of 10,000,000 simulation runs, we have found that the model only exhibits this behavior approximately 25% of the time. Therefore, the correct conclusion to be drawn by the algorithms should be the model does not exhibit this behavior more than 28% of the time.

Assume a user wants the error rate to be around 1% (*α *= 0.01 *β *= 0.01) and has chosen *δ *to be 0.05. This time, there is a 5.4% probability that Younes A gives a wrong conclusion while there is a 25.4% probability that Younes B gives a wrong or undecided conclusion, whereas there is only a 0.5% probability that OSM A and OSM B make a wrong conclusion (Table 1). On the other hand, if the user had chosen a smaller *δ *( = 0.025), they would have been able to control the error rates (Table 1). Therefore, one naive strategy would be to always choose an extremely small *δ *that is close to 0. However, since the expected number of samples of Younes A and Younes B are inversely proportional to *δ*^2 ^[13], such a strategy would have required an exorbitant number of simulation runs.

In the two scenarios above, we have chosen different values of *δ *for Younes A and Younes B. Unfortunately, it was insufficient in both cases, causing Younes A and Younes B to not perform well (i.e. keeping error rates under control). This clearly shows that their success or failure depends heavily upon the value of *δ *and, in practice, it is unrealistic to expect users to be able to provide a suitable *δ *for every scenario. Therefore, eliminating the need for users to decide on the value *δ*, and dynamically selecting the optimal value depending on the situation, is a useful practical solution as proposed in OSM A and OSM B.

## Discussion

In this paper, we have presented two algorithms (OSM A and OSM B) that are similar but serve different purposes. OSM A is recommended when computational resources are plentiful and/or bounding the error rates is a priority. In the situation where computational resources are limited, OSM B is useful. While these algorithms are founded upon a simple idea, the improvements over current state-of-the-art algorithms are significant and practically useful. Firstly, our algorithms do not require the critical, yet difficult to determine indifference region as an input parameter. Secondly, our algorithms adjust automatically to the difficulty of the problem by dynamically halving the indifference region, leading to using fewer samples when *p *is far away from *θ*. Lastly, it always returns a definite response to the user, which is either guaranteed to be error bounded given sufficient samples or comes with a confidence measure if computational resources are limited.

Therefore, we foresee the usage of these algorithms to be wide as there is no assumption or requirement of the simulation model, allowing them to be applied to any stochastic system analysis.

## Competing interests

The authors declare that they have no competing interests.
